# Predictors of Longitudinal Viral Load count and Survival Time to Death Among Adult TB/HIV Coinfected Patients Treated at Two Selected Amhara Region Comprehensive Specialized Hospitals, Ethiopia

**DOI:** 10.1002/hsr2.70867

**Published:** 2025-06-17

**Authors:** Nurye Seid Muhie, Abdela Assefa Bekele, Awoke Seyoum Tegegne

**Affiliations:** ^1^ Department of Statistics Mekdela Amba University Tulu Awulia Ethiopia; ^2^ Department of Statistics Assosa University Assosa Ethiopia; ^3^ Department of Statistics Bahir Dar University Bahir Dar Ethiopia

**Keywords:** association, Bahir Dar, coinfected, death, Gondar, HIV, predictors, TB, viral load

## Abstract

**Background and Aims:**

The most prevalent opportunistic illness among people living with HIV/AIDS is tuberculosis. The aim of this study was to determine predictors of longitudinal viral load and survival time to death among adult TB/HIV coinfected patients treated at two selected Amhara region Comprehensive Specialized Hospitals, Ethiopia.

**Methods:**

A retrospective follow‐up study design was conducted from March 2018–2022 in the University of Gondar Comprehensive Specialized Hospital and Felege Hiwot Comprehensive Specialized Hospital. Descriptive statistics, separate Cox PH model, separate generalized linear mixed model, and joint model were employed to analyze the coinfected patient data.

**Results:**

Among 253 TB/HIV coinfected participants, 26.5% mortality and the rest were censored. Random intercept and slope model for the longitudinal viral load count Cox PH model for time to death were selected based on AIC and BIC values. The estimate of the association parameter due to the slope ($\gamma_{1}=0.4981)$ and due to the viral load count variability through time is positive ($\gamma_{2}=0.6247$).

**Conclusions:**

These results concluded that the joint model is not only the simplest model, but also provided a better fit to the coinfected patients' data than the separate model. The parameter estimation under the joint model revealed that INH, residence, CD4 cell count, functional status, and BMI were considered as significant joint predictors of viral load count and time to death among TB/HIV coinfected patients. Furthermore, the results of the association parameter concluded that the higher the viral load count of the patient, the higher the chance of mortality, and correspondingly, patients with lower viral load count have a lower chance of mortality. In this study, important potential joint predictors should be given special attention by adult TB/HIV coinfected patients and health professionals to minimize viral load and risk of mortality.

**Abbreviations**
AIC: Akaike information criteria
ART: antiretroviral therapy
BIC: Bayesian information criterion
BMI: body mass index
CD4: cluster differentiation 4
CPT: cotrimoxazole preventive therapy
FHCSH: Felege Hiwot comprehensive specialized hospital
GLMEM: generalized Linear mixed effect model
HIV: Human Immune Deficiency virus
INH: isoniazid acid hydrazide
OCC: other comorbid condition
OIs: opportunistic infections
PHs: proportional hazards
RBC: red blood cell
TB: tuberculosis
UGCSH: University of Gondar comprehensive specialized hospital
WBC: White Blood Cell
WHO: World Health Organization

## 1 Background

The most prevalent opportunistic illness among people living with HIV/AIDS is tuberculosis (TB), and because of the mutually reinforcing nature of the two infections, people who have both infections are at a significantly increased risk of dying [1]. Taking into consideration viral load count and anti‐retroviral therapy (ART), the risk of death for coinfected individuals is about twice that of HIV‐positive individuals alone. High viral loads and patients' immature immune systems increase the likelihood of fast TB/HIV development, and high mortality has been realized in TB and HIV coinfection [2, 3].

An estimated 9.6 million new cases and 1.5 million fatalities from TB were reported in 2018; the bulk of these cases and deaths occurred in developing nations, primarily in Asia and Africa. Additionally, there were 390,000 deaths from TB/HIV, with an estimated 1.2 million cases of HIV coinfection [4].

The bidirectional relationship between HIV and TB has been found by retrospective cohort and case‐control studies carried out in several regions of Ethiopia. For instance, a nationwide retrospective cohort study found that 9.0% of HIV patients receiving treatment also had TB coinfection. Another study carried out in Ethiopia's Amhara region found that 27.7% of HIV individuals on ART also got tuberculosis. However, according to a study done in Bahirdar, 30.6% of TB patients also tested positive for HIV, and more than 25% of TB‐HIV cases were also deaths [5, 6, 7].

In addition, predictors significantly associated with time to death among coinfected with TB and HIV were Baseline Body Mass Index (BMI), Extra‐pulmonary TB, Coinfected with Latent TB infection, completing TB treatment, Cotri‐moxazole Prophylactic Therapy (CPT) [8], residence, substance use, educational status, INH drug users [9], age over 50 years, CD4+ T lymphocyte count ≤ 200 cells/mm^3^, detectable viral load, and non‐use of antiretroviral therapy [10], marital status, antiretroviral treatment (ART) regimen, religion, sqrt CD4 cell [11], ages between 25 and 34 years, patients with OCC, and substance users, sex, adherence status, baseline hemoglobin [12], year in ART enrollment, aged between 35 and 44 years, functional status, and WHO stage‐II and IV [13].

Similarly, predictors like older age, underweight, and past opportunistic infection [14], history of HIV screening test, and diagnosis‐treatment interval [15], urban residence, CD4 count of 201–500 cells/mm^3^ and long duration on ART [16], poor adherence [17], patient substance use, visit time, baseline hemoglobin, and the interaction of visit time by substance use [18], primary educators, patients disclosed the disease to family member, viral load < 10,000 copies/mL, hemoglobin level ≥ 11 g/dL, CD4 cell count ≥ 200 per mm^3^, weight ≥ 50 kg, BMI between 18.5 and 24.9 kg/m^3^, fair and good treatment adherence, advanced WHO clinical stages, patients with OCC, and substance use [19], individuals with fair adherence were a significant predictor variables for viral load count at 5% level of significance.

However, there were no previous studies that were done jointly, as mentioned above, on the time to death and viral load count among adult coinfected patients. Consequently, filling this knowledge gap enables public health professionals to develop effective intervention measures to stop the disease and better assist/understand the relationship between death and viral load count. This study will also bridge the information gap and update the previous knowledge of the same problem. Therefore, the objective of this study was to determine predictors of longitudinal viral load and survival time to death among adult TB/HIV coinfected patients treated at two selected Amhara region Comprehensive Specialized Hospitals, Ethiopia.

## 2 Methods

### 2.1 Study Area and Design

A retrospective follow‐up was conducted at the University of Gondar Comprehensive Specialized Hospital (UGCSH) and Felege Hiwot Comprehensive Specialized Hospital (FHCSH), Ethiopia.

### 2.2 Study Period

This retrospective follow‐up study spanned between March 2018 and March 2022 for both UGCSH and FHCSH.

### 2.3 Inclusion Criteria

TB/HIV coinfected patients whose age was greater than or equal to 15 years who received ART were included as the study participants. Coinfected patients who had at least two follow‐up visits for viral load during the study period were eligible. Patients under the study period were also included in this study.

### 2.4 Exclusion Criteria

Coinfected patients who were lost to follow‐up, dropped, transferred out to other nearest clinics, respectively, for both cases, and died due to other causes were considered as exclusion criteria. In addition, coinfected patients who had only one follow‐up visit for viral load, patients who received treatment for only TB or HIV, and patients without study period were considered as exclusion criteria.

### 2.5 Procedure of Sample Size Determination

**Figure 1:**
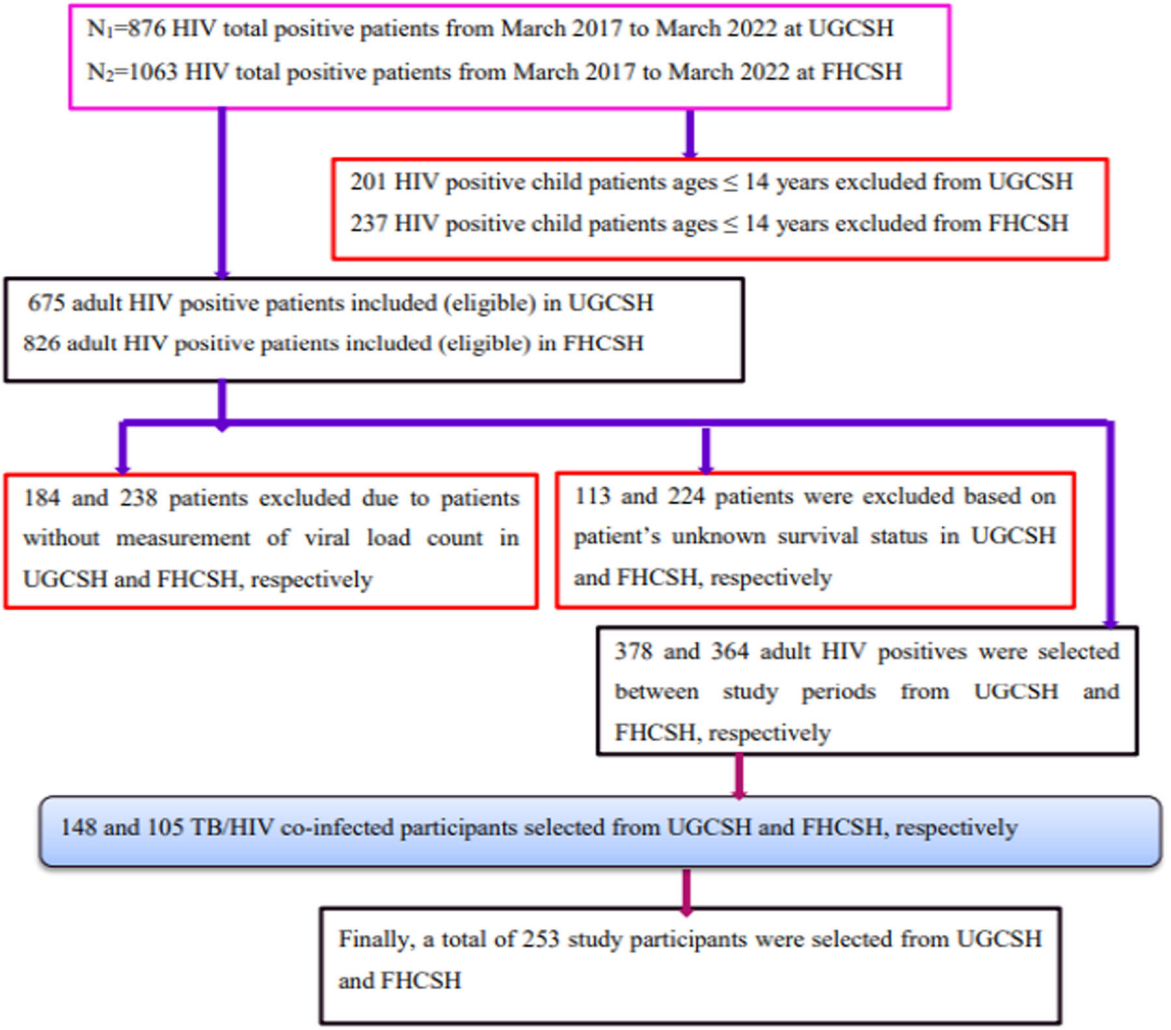
Conceptual framework sample size determination procedure.

The current study included 253 TB/HIV coinfected patients between March 2018 and the 2022 treatment period in UGCSH and FHCSH. Based on inclusion and exclusion criteria, 253 adult coinfected patients were selected (Figure 1).

### 2.6 Response Variables

Viral load count and survival time to death of TB/HIV coinfected patients were considered as response variables.

### 2.7 Predictor Variables

Potential predictor variables that might have an effect on viral load count and survival time to death of coinfected patents were baseline viral load count, gender, age in years, residence, level of education, disclosure status, a cluster of differentiation 4 (CD4) count per mm^3^, hemoglobin level per dL, weight in kg, body mass index (BMI) in kg/m^2^, WHO clinical stage, adherence, functional status, Tuberculosis (TB) Status, and Opportunistic infection (OIs) other than TB, isoniazid or isonico‐tinic acid hydrazide (INH), cotrimoxazole prophylactic therapy (CPT), and substance addiction were the predictor variables.

### 2.8 Statistical Model

A generalized linear mixed effect model (GLMEM) and Cox PHs model were used in this study to analyze longitudinal and survival data, respectively. Before joint model analysis, separate longitudinal and survival models were applied to determine the separate predictors for viral load and time to death. The AIC and BIC were used to determine which separate survival, longitudinal model is more parsimonious, the covariance structure for longitudinal response, and the comparison of the separate model with the joint model.

### 2.9 Methods of Missing Data Mechanisms

The nature of the data for this study was longitudinal; some data information was expected to be missing. To eradicate this problem, multiple imputations of missing data mechanisms are utilized, which take into account the ambiguity caused by missing data information and produce a valid statistical inference.

### 2.10 Methods of Variable Selection

In this study, we used purposeful variable selection methods [9, 19] at a 25% level of significance for viral load count and time to death.

Generally in this method section, statistical tests are used in hypothesis testing and they can be used to determine whether a predictor variable has a statistically significant relationship with an outcome variable. We used a 5% priori level of significance to determine whether or not the predictor variables have an effect on response variables. R statistical software package(s) version 4.4.2 were used to analysis patient's data.

### 2.11 Ethics Approval and Consent to Participate

All methods were performed by the ethical standards as laid down in the Declaration of Helsinki. This study was approved by the Bahir Dar University Research Technical and Ethical Review Board (Ref. no Stat‐ S/164/166/2022). The informed consent was waived by the Bahir Dar University Research Technical and Ethical Review Board.

## 3 Results

In this study, some missing data is managed using SPSS version 27 through multiple imputations. After multiple imputations of missing data mechanisms, the following results would be concentrated for our study.

### 3.1 Sociodemographic and Clinical Predictors' Descriptive Results

Tables 1 and 2 indicate the descriptive section of predictor categories with survival time and total participants.

**Table 1:** Baseline clinical characteristics of TB/HIV coinfected patients.

Variable	Categories	Number of death (%)	Number of censored (%)	Total (%)
Weight	< 50 kg	29 (21.0)	109 (79.0)	138 (54.5)
	≥ 50 kg	38 (33.0)	77 (67.0)	115 (45.5)
Body mass index	< 18.5	27 (26.5)	75 (73.5)	102 (40.3)
	18.5–24.9	23 (29.1)	56 (70.9)	79 (31.2)
	≥ 25	17 (23.6)	55 (76.4)	72 (28.5)
CD4 cell count	< 200 cells/mm^3^	41 (29.9)	96 (70.1)	137 (54.2)
	> = 200 cells/mm^3^	26 (22.4)	90 (77.6)	116 (45.8)
Viral load count	> = 10,000	46 (26.7)	126 (73.3)	172 (68.0)
	< 10,000	21 (25.9)	60 (74.1)	81 (32.0)
Adherence	Poor	29 (55.8)	23 (44.2)	52 (20.5)
	Fair	26 (40.0)	39 (60.0)	65 (25.7)
	Good	12 (8.8)	124 (91.2)	136 (53.8)
OIs	No	31 (16.4)	158 (83.6)	189 (74.7)
	Yes	36 (56.3)	28 (43.7)	64 (25.3)
OCC	No	23 (11.9)	171 (88.1)	194 (76.7)
	Yes	44 (74.6)	15 (625.4)	59 (23.3)
CPT	No	42 (28.8)	104 (71.2)	146 (57.7)
	Yes	25 (23.4)	82 (76.6)	107 (42.3)
INH	No	39 (29.3)	94 (70.7)	133 (52.6)
	Yes	28 (23.3)	82 (68.3)	120 (47.3)
ART regimen	1 d	20 (30.8)	15 (69.2)	65 (25.7)
	1c	17 (28.8)	42 (71.2)	59 (23.3)
	1e	14 (22.6)	48 (77.4)	62 (24.5)
	Others	18 (26.9)	49 (73.1)	67 (26.5)
WHO clinical stage	Stage‐I	13 (12.9)	88 (87.1)	101 (33.9)
	Stage‐II	15 (24.6)	46 (75.4)	61 (24.1)
	Stage‐III	21 (45.7)	25 (54.3)	46 (18.2)
	Stage‐IV	18 (40.0)	27 (60.0)	45 (17.8)
Types of TB	Pulmonary	26 (17.9)	119 (82.1)	145 (57.3)
	Extra‐pulmonary	41 (38.0)	67 (62.0)	108 (42.7)
Total	—	67 (26.5)	186 (73.5)	253 (100)

**Table 2:** Baseline sociodemographic characteristics of TB/HIV coinfected patients.

Variable	Categories	Number of deaths (%)	Number of censored (%)	Total (%)
Gender	Female	39 (25.0)	117 (75.0)	156 (61.7)
	Male	28 (28.9)	69 (71.1)	97 (38.3)
Age	15–24	18 (41.9)	25 (58.1)	43 (17.0)
	25–34	22 (26.8)	60 (73.2)	82 (32.4)
	35–44	14 (25.0)	42 (75.0)	56 (22.1)
	> 44	13 (18.1)	59 (81.9)	72 (28.5)
Residence	Rural	38 (32.8)	78 (67.2)	116 (45.8)
	Urban	29 (21.2)	108 (78.8)	137 (54.2)
Level of education	Non‐educated	23 (29.1)	56 (70)	79 (31.2)
	Primary	18 (24.7)	55 (75.3)	73 (28.9)
	Secondary	15 (22.4)	52 (77.6)	67 (26.5)
	Tertiary	11 (32.4)	23 (67.6)	34 (13.4)
Disclosure status	No	43 (68.3)	20 (31.7)	63 (24.9)
	Yes	24 (12.6)	166 (87.4)	190 (75.1)
Substance use	No	38 (19.8)	154 (80.2)	192 (75.9)
	Yes	29 (47.5)	24 (52.5)	61 (24.1)
Total	—	67 (26.5)	186 (73.5)	253 (100)

Most of the patients (54.5%) weighed < 50 kg, and about 21% of patients weight < 50 kg died from the disease. Approximately less than half of the patients (40.3%) had a body mass index < 18.5 kg/m^2^, and consequently, 26.5% of patients in these categories died. About 68.0% of patients had unsuppressed viral load at baseline, and 26.7% had died. More than half of the patients (53.8%) were good treatment adherents, and a lower percentage of deaths (8.8%). Similarly, more than half of the patients (75% and 77%) were without OIs and OCC, and a lower percentage of deaths (16.4% and 11.9%), respectively. More than 50% of patients were CPT users, INH users, pulmonary, and TB. Around one‐fourth (25%) of patients were on 1‐day ART regimens and Clinical stage I. Finally, the overall death rate of TB/HIV coinfected patients was 26.5% (Table 1).

Among the total 253 study participants that were considered in this study, about 61.7% were female, and among them, 25.0% of female patients died. Nearly 32% of patients were between 25 and 34 years old, and among them, 26.8% lived in an urban area and died from disease. About more than half of the patients (54%) lived in urban areas, and 21.2% of urban patients died due to TB/HIV. Around 29% were primary education, and 75% disclosed the disease to a family member. Similarly, around 76% of patients were non‐substance addicted, and among them, 19.8% died from TB/HIV (Table 2).

### 3.2 Selection of Covariance Structure, Random Effect GLMEM, and Survival Model

Unstructured covariance (UN) structure, random intercept, and slope model for the longitudinal viral load count were selected based on AIC and BIC values. Similarly, the semiparametric Cox PH model was selected by considering the lowest criterion values (Table 3).

**Table 3:** Comparison of covariance structure, random effect model, and survival model.

	AIC	BIC
Covariance Structure		
AR(1)	902.947	1050.043
UN	901.198	1034.052
CS	903.583	1068.299
Model for random effect		
Random intercept	1072.943	1089.3
Random slope	989.139	1034.002
Random intercept and slope	901.098	1013.673
Survival model		
Cox PHs	854.2626	926.387
Weibull	856.5929	972.9761
Exponential	1025.331	1139.951
Log logistic	887.8726	1006.586
Log normal	910.1715	1028.885

### 3.3 Purposeful Variable Selection for Viral Load Count and Time to Death

The predictors gender, INH, CPT, disclosure, CD4 cell count, viral load count, residence, weight, BMI, WHO, TB type, functional status, OIs, and substance use were significant impact factors at a 25% level of significance for time to death. However, the remaining predictors were not significant impact factors for time to death.

In a similar way, predictors age, educational level, INH, CPT, Hgb level, CD4 cell count, viral load count, weight, BMI, adherence, residence, TB type, and functional status use were significant impact factors at a 25% level of significance for viral load count. However, the remaining predictors were not significant impact factors for viral load.

These results also indicated that the result of AIC and BIC for the joint model was smaller than the separate model (Table 4).

**Table 4:** AIC and BIC results for comparison of the separate and joint models.

	AIC	BIC
Separate model		
Cox PHs	713.8305	837.493
Random intercept and slope	861.360	936.394
Joint model		
Cox PHs	678.0841	812.1784
Random intercept and slope	835.4871	903.6354

Joint model was more robust with regard to parameter estimation and standard errors than the separate model (Tables 5–, 7). Hence, the joint model is not only the simplest model, but also provided a better fit to the coinfected patients data. In this study the overall interpretation, discussion, conclusion, and recommendations were based on joint model.

**Table 5:** Parameter estimation for the Cox PH model time to death.

Variables	Categories	Estimate (β)	Std. error	*p* value	HR	95% CI for HR
Gender (ref: female)	Male	0.9898	0.1442	0.045*	2.6908	(2.0282, 3.5698)
INH (ref: no)	Yes	−0.9429	0.4597	0.040*	0.3895	(0.1582, 0.9590)
CPT (ref: no)	Yes	−0.8618	0.4276	0.044*	0.4224	(0.0240, 0.4740)
Disclosure (ref: no)	Yes	−1.2590	0.4421	0.004*	0.2839	(0.1194, 0.6754)
CD4 count/mm^3^ (ref: < 200)	> = 200	−0.3373	0.1253	0.036*	0.7137	(0.5583, 0.9124)
Viral load (ref: > = 10,000)	< 10,000	−0.4351	0.2179	< 0.001*	0.6472	(0.0081, 0.3680)
Residence (ref: rural)	Urban	−0.2353	0.0824	0.026*	0.2653	(0.6725, 0.9289)
Weight (ref: < 50)	> = 50 kg	−0.9234	0.3126	0.043*	0.3972	(0.2152, 0.7330)
BMI (ref =underweight)	Normal	−0.3301	0.2852	> 0.99	0.2469	(0.7955, 2.4328)
	Obesity	0.3435	0. 0164	0.0067*	1.4099	(1.3653, 1.4559)
Functional status (ref=working)	Ambulatory	0.5436	0.3636	0.135	1.7221	(0.8444, 3.5124)
	Bedridden	2.3569	0.4291	< 0.001*	10.559	(4.5536, 24.484)
WHO (ref: stage‐I)	Stage‐II	0.1256	0.5021	0.802	1.1339	(0.4238, 3.0339)
	Stage‐III	0.2558	0.5360	0.633	1.2914	(0.4516, 3.6928)
	Stage‐IV	−1.0586	0.5779	0.067	0.3469	(0.1118, 1.0771)
TB type (ref: no)	Extra	0.4777	0.2011	< 0.001*	1.6124	(1.0870, 2.3916)
OIs (ref: no)	Yes	0.0131	0.3430	> 0.99	0.9870	(0.5039, 1.9332)
Substance use (ref: no)	Yes	0.4537	0.5446	0.635	0.4047	(0.2185, 1.8471)

*Note:* Std. error represents standard error, HR indicates hazard ratio of estimates, and CI indicates confidence interval for hazard ratios. *p* values < 0.001, indicate “*p *< 0.001”; for *p* values between 0.001 and 0.01, indicate the value to the nearest thousandth; for *p* values ≥ 0.01, indicate the value to the nearest hundredth; and for *p* values > 0.99, indicate as “*p *> 0.99”.

* is statistically significant at 5% level of significance.

**Table 6:** Parameter estimation for the generalized linear mixed effect model viral load count.

Variables	Categories	Estimate(β)	Std. error	*p* value
Intercept	—	5.7763	0.2472	< 0.000*
Visit time	—	−0.2542	0.0031	< 0.001*
Age (ref: 15–24)	25–34	0.5986	0.1801	< 0.001*
	35–44	0.4045	0.1306	0.027*
	> 44	0.2579	0.1650	0.121
Educational level (ref = non‐educated)	Primary	0.2579	0.1650	0.211
	Secondary	−0.0992	0.1472	0.502
	Tertiary	0.2169	0.1562	0.166
Hgb level (ref: no)	Yes	0.2144	0.0701	0.048*
Viral load (ref: > = 10,000)	< 10,000	−1.7134	0.1139	< 0.000*
CD4vcount/mm^3^ (ref: < 200)	> = 200	−1.0420	0.1013	0.0058*
Weight (ref: < 50)	> = 50 kg	−1.3014	0.1933	0.0081*
BMI (ref = under‐weight)	Normal	−0.9021	0.1217	0.041*
	Obesity	0.8281	0.1479	0.038*
Functional status (ref: working)	Ambulatory	0.3015	0.1301	0.028*
	Bedridden	0.0304	0.1893	0.873
Residence (ref: rural)	Urban	−0.3596	0.1425	0.013*
INH (ref: no)	Yes	−0.9019	0.0250	0.0038*
CPT (ref: no)	Yes	0.1950	0.3212	0.8087
Adherence use (ref: poor)	Fair	−0.0219	0.1489	0.8835
	Good	−0.2354	0.0601	0.0096*
TB type (ref: pulmonary)	Extra pulmonary	0.7037	0.1028	0.045*

*Note:* Std. error represents standard error. *p* values < 0.001, indicate “*p *< 0.001”; for *p* values between 0.001 and 0.01, indicate the value to the nearest thousandth; for *p* values ≥ 0.01, indicate the value to the nearest hundredth; and for *p* values > 0.99, indicate as “*p *> 0.99”.

* is statistically significant at 5% level of significance.

**Table 7:** Parameter estimation for the joint Cox PH model and generalized linear mixed effect.

Variables	Categories	Survival sub model			Longitudinal sub model		
		Estimate (β)	Std. error	*p* value	Estimate (β)	Std. error	*p* value
Intercept	—	—	—	—	5.9810	0.4683	< 0.001*
Gender (ref: female)	Male	0.9984	0.1442	0.0980	—	—	—
Age (ref: 15–24)	25–34	—	—	—	0.2864	0.1921	0.114
	35–44	—	—	—	0.4326	0.2917	0.068
	> 44	—	—	—	0.1457	0.1260	0.135
Educational level (ref = non‐educated)	Primary	—	—	—	0.3481	0.1761	0.154
	Secondary	—	—	—	−0.0841	0.1583	0.612
	Tertiary	—	—	—	0.3074	0.2134	0.176
INH (ref: no)	Yes	−0.9638	0.3485	0.020*	−0.8042	0.1347	0.043*
CPT (ref: no)	Yes	−0.8798	0.4175	0.0048*	‐0.2842	0.3018	0.108
Disclosure (ref: no)	Yes	−1.1472	0.4012	0.04*	—	—	—
Residence (ref: rural)	Urban	−0.3583	0.1714	0.048*	−0.3454	0.0161	0.015*
Hgb level (ref: < 11)	> = 11	—	—	—	0.1346	0.1814	0.258
CD4 count/mm^3^ (ref: < 200)	> = 200	−0.2463	0.1042	0.047*	−0.5042	0.1240	0.016*
Viral load (ref: > = 10,000)	< 10,000	−0.1087	0.1179	0.102	−0.7238	0.1249	0.0021*
Weight (ref: < 50)	> = 50 kg	−0.8657	0.2135	0.054	−0.0147	0.1247	0.059
BMI (ref = under‐weight)	Normal	−0.5461	0.2453	1.392	−0.1278	0.1845	0.461
	Obesity	0.3456	0.0152	0.0047*	0.8394	0.1584	0.0020*
Functional status (ref = working)	Ambulatory	0.6137	0.3636	0.135	0.1023	0.1481	0.430
	Bedridden	2.4697	0.0180	0.015*	0.3502	0.0974	0.0030*
WHO (ref: Stage‐I)	Stage‐II	0.1061	0.4110	0.915	—	—	—
	Stage‐III	0.2435	0.4254	0.743	—	—	—
	Stage‐IV	−0.0350	0.4667	0.086	—	—	—
Adherence use (ref: poor)	Fair	—	—	—	−0.0212	0.1547	0.965
	Good	—	—	—	−0.1256	0.1784	0.488
TB type (ref: pulmonary)	Extra pulmonary	0.5146	0.2015	< 0.001*	0.1034	0.1687	0.104
OIs (ref: no)	Yes	0.0378	0.2320	0.768	—	—	—
Substance use (ref: no)	Yes	0.3486	0.4320	0.758	—	—	—
Visit time	—	—	—	—	−0.2143	0.0041	< 0.001*
$\gamma_{1}$	—	0.4981	0.0143	< 0.001*	—	—	—
$\gamma_{2}$	—	0.6247	0.0813	0.013*	—	—	—

*Note:* Std. error represents standard error. $\gamma_{1}$ and $\gamma_{2}$ are the association parameters under survival sub model. *p* values < 0.001, indicate “*p *< 0.001”; for *p* values between 0.001 and 0.01, indicate the value to the nearest thousandth; for *p* values ≥ 0.01, indicate the value to the nearest hundredth; and for *p* values > 0.99, indicate as “*p *> 0.99”.

* is statistically significant at 5% level of significance.

### 3.4 The Separate Survival and Longitudinal Analysis Results for Time to Death and Viral Load

The separate model also carried out for time to death exposed that, among the potential predictor variables gender, INH, CPT, disclosure of the disease to family member, CD4 cell count, baseline viral load count, weight, BMI, functional status, residence, and TB type were determined as a significant predictors related to survival time to death (Table 5).

Based on the results of separate GLMEM analysis for viral load count, visit time, hemoglobin level, baseline viral load, CD4 cell count, weight, BMI, functional status, residence, INH, adherence, residence, and TB type were factors that significantly affected longitudinal viral load count (Table 6).

### 3.5 Joint Model Analysis Result for Time to Death and Viral Load

The joint model for longitudinal and survival joint model result showed that INH, residence, CD4 cell count, functional status, and BMI were considered as a significant joint determinant factors of viral load count and time to death among TB/HIV coinfected patients (Table 7).

The result of Table 7 also indicated, the average number of viral load count of patients who took INH drug was decreased by 0.8 copies/mL as compared to INH drug non‐users (β=−0.8042,pvalue=0.0430) and correspondingly the hazard of death for INH drug users was decreased by 61.8% than non‐users patient, keeping the other predictors constant (exp⁡(β)=0.3814,pvalue=0.0203).

Similarly, as compared to rural patients, the average no of viral load count for urban patients was decreased by 0.3 copies/mL (β=−0.3454,pvalue=0.0153) and correspondingly, the estimated hazard of death for urban patients was decreased by 30% than rural patients, keeping the other predictors constant (exp(β)=0.6989,pvalue=0.0478).

The current study also revealed that, as compared to baseline CD4 cell count ≥ 200 cells/mm^3^ with < 200 cells/mm^3^, coinfected patient's baseline CD4 cell count was increased by one unit, and the average viral load count decreased by 0.5 copies/mL (β=−0.0542,pvalue=0.0160). Similarly, the baseline CD4 cell count of coinfected patient increased, the current hazard of death of the patients whose CD4 cell count ≥ 200 cells/mm^3^ was decreased by 21.8% than CD4 cell < 200 cells/mm^3^, keeping the other predictors constant (exp(β)=0.7817,pvalue=0.0478).

On the other hand, bedridden coinfected patients had an increased average viral load count by 0.35 copies/mL than working patients (β=0.3502,pvalue=0.0030) and correspondingly, the hazard of death for bedridden patients was increased by 59.9% than working patients, keeping the other predictors constant (β=1.5995,pvalue=0.0150).

Table 7 also indicated that when obesity BMI patients were compared with underweight patients, the average viral load count for obesity patients was increased by 0.8 copies/mL (β=0.8394,pvalue=0.0020) and correspondingly, the hazard of death for obesity patients was increased by 41.3% than underweight patients, keeping the other predictors constant (exp(β)=1.4128,pvalue=0.0047).

The estimate of the association parameter due to the slope of viral load count is positive ($\gamma_{1}=0.4981)$. This implies that the slope of viral load count is positively associated with the hazard of death. Similarly, the estimate of the association parameter due to the viral load count variability is also positive ($\gamma_{2}=0.6247$) which represents that the higher viral load count is associated with the higher hazard of death (Table 7).

## 4 Discussion

The estimate of the association parameter indicates that the slope of viral load count and viral load count variability is positively associated with the hazard of death. This implies, the higher viral load count is associated with the higher hazard of death. The clinical implications of this finding is patients who had higher amount of viral load concentrations leads to a higher chance of mortality. The result of this study is consistent with previous studies [14, 15, 18, 19].

The result of this study showed that patients who had taken INH drug, leads to a weakened virus amount, consequently causing less hazard of death. This result is in line with previous studies [20, 21]. However, this result is contradicted by a study done in northwest Ethiopia [22]. The results expressed that INH‐treated TB/HIV coinfected patients had a 5.21‐fold longer survival time to death than nontreated TB/HIV coinfected patients. However, this result is inconsistent with the previous result [9].

The result of this study also found that the average viral load count and time to death of coinfected patients in urban areas decreased with visit time as compared to rural coinfected patients. This could be attributed to urban area patients having better follow‐ up of treatment, which causes viral load count to obviously decrease. On the other hand rural patients have a high increment of average viral load count and time to death, the major reasons may be, weight loss, poor treatment setting, high distance from treatment center, higher cost of transportation, less awareness of medication, fear of stigma and discrimination related to the community. This result is in line with previous studies [23, 24]. Contrariwise, this result is opposed to the previous result [22, 25].

The patient's baseline CD4 cell count is a significant predictor of the variable of interest. This result was consistent with studies [26]. Similarly, as the baseline CD4 cell count of coinfected patients increased, the current hazard of death of the patients decreased. This result is supported by other research [21, 27, 28]. The reason for this might be that such patients started their treatment before an increase in viral load count, and treatment decreases the viral load count and hazard of death.

Bedridden coinfected patients had a higher viral load count than working patients. The reason for this is that coinfected patients who are bedridden have poor treatment, which could be because they are more aware of the disease's difficulty and are more likely to develop opportunistic disease. So, patients may be unable to work and care for themselves, require different levels of assistance from families or others, live at home, which may include sleeping in a bedroom, and may lead to death due to disease progression. Our finding is supported by previous research [12, 22, 29, 30, 31]. However, this idea is inconsistent with a previous study [7], which indicated that one of the independent predictors of mortality during TB/HIV treatment was ambulatory rather than bedridden coinfected patients.

The average viral load count and the corresponding hazard of death for obesity patients were higher than underweight patients. The result of these studies are consistent with previous studies [14, 19, 32]. Conversely, the result of this study contradicts with previous result [33, 34].

## 5 Conclusion and Recommendation

These results concluded that the joint model is not only the simplest model, but also provided a better fit for the coinfected patients' data than the separate model. The parameter estimation under the joint model revealed that INH, residence, CD4 cell count, functional status, and BMI were considered as significant joint predictors of viral load count and time to death among TB/HIV coinfected patients. Furthermore, the results of the association parameter concluded that the higher the viral load count of the patient, the higher the chance of mortality, and correspondingly, patients with lower viral load count have a lower chance of mortality.

In this study, important potential joint predictors should be given special attention by adult TB/HIV coinfected patients and health professionals to minimize viral load and risk of mortality. Health professionals should perform health issue studies to enhance public awareness about the predictors that are related to viral load count and risk of death. The health professionals also recommend to the concerned bodies that TB/HIV medication must be reasonable and accessible to coinfected patients to minimize viral load count and death, and to live with better health outcomes.

## Author Contributions

**Nurye Seid Muhie:** conceptualization, methodology, software, data curation, investigation, validation, formal analysis, supervision, writing original draft, visualization, writing – review and editing, and resources. **Abdela Assefa Bekele:** conceptualization, methodology, data curation, investigation, validation, supervision, writing original draft, visualization, and writing – review and editing. **Awoke Seyoum Tegegne:** conceptualization, writing – review and editing of final manuscript.

## Conflicts of Interest

The authors declare no conflicts of interest.

## Transparency Statement

The lead author Nurye Seid Muhie affirms that this manuscript is an honest, accurate, and transparent account of the study being reported; that no important aspects of the study have been omitted; and that any discrepancies from the study as planned (and, if relevant, registered) have been explained.

## Data Availability

The authors confirm that the data supporting the findings of this study are available from the corresponding author upon reasonable request.
